# GPR34 in spinal microglia exacerbates neuropathic pain in mice

**DOI:** 10.1186/s12974-019-1458-8

**Published:** 2019-04-11

**Authors:** Akira Sayo, Hiroyuki Konishi, Masaaki Kobayashi, Kuniyuki Kano, Hiroki Kobayashi, Hideharu Hibi, Junken Aoki, Hiroshi Kiyama

**Affiliations:** 1 Department of Functional Anatomy and Neuroscience, Nagoya University Graduate School of Medicine, 65, Tsurumai-cho, Showa-ku, Nagoya, 466-8550 Japan; 2 Department of Oral and Maxillofacial Surgery, Nagoya University Graduate School of Medicine, Nagoya, 466-8550 Japan; 3 Department of Molecular and Cellular Biochemistry, Tohoku University Graduate School of Pharmaceutical Sciences, Sendai, 980-8578 Japan; 4 Department of Pathology, Nagoya University Graduate School of Medicine, Nagoya, 466-8550 Japan

**Keywords:** Allodynia, GPCR, GPR34, LysoPS, Microglia, Neuroinflammation, Pain

## Abstract

**Background:**

Neuropathic pain is caused by sensory nerve injury, but effective treatments are currently lacking. Microglia are activated in the spinal dorsal horn after sensory nerve injury and contribute to neuropathic pain. Accordingly, molecules expressed by these cells are considered potential targets for therapeutic strategies. Our previous gene screening study using a mouse model of motor nerve injury showed that the G-protein-coupled receptor 34 gene (GPR34) is induced by nerve injury. Because GPR34 is now considered a microglia-enriched gene, we explored the possibility that it might be involved in microglial activation in the dorsal horn in a mouse model of neuropathic pain.

**Methods:**

mRNA expression of GPR34 and pro-inflammatory molecules was determined by quantitative real-time PCR in wild-type and GPR34-deficient mice with L4 spinal nerve injury. In situ hybridization was used to identify GPR34 expression in microglia, and immunohistochemistry with the microglial marker Iba1 was performed to examine microglial numbers and morphology. Mechanical sensitivity was evaluated by the von Frey hair test. Liquid chromatography–tandem mass spectrometry quantified expression of the ligand for GPR34, lysophosphatidylserine (LysoPS), in the dorsal horn, and a GPR34 antagonist was intrathecally administrated to examine the effect of inhibiting LysoPS-GPR34 signaling on mechanical sensitivity.

**Results:**

GPR34 was predominantly expressed by microglia in the dorsal horn after L4 nerve injury. There were no histological differences in microglial numbers or morphology between WT and GPR34-deficient mice. However, nerve injury-induced pro-inflammatory cytokine expression levels in microglia and pain behaviors were significantly attenuated in GPR34-deficient mice. Furthermore, the intrathecal administration of the GPR34 antagonist reduced neuropathic pain.

**Conclusions:**

Inhibition of GPR34-mediated signal by GPR34 gene deletion reduced nerve injury-induced neuropathic pain by suppressing pro-inflammatory responses of microglia without affecting their morphology. Therefore, the suppression of GPR34 activity may have therapeutic potential for alleviating neuropathic pain.

**Electronic supplementary material:**

The online version of this article (10.1186/s12974-019-1458-8) contains supplementary material, which is available to authorized users.

## Background

Neuropathic pain is often caused by nerve injury resulting from surgery, diabetes, bone compression, or infection [1]. Neuropathic pain includes tactile allodynia, which is pain caused by a non-noxious stimulus, and thermal hyperalgesia, an augmented pain response to noxious heat or cold. Currently available treatments such as nonsteroidal anti-inflammatory drugs and opioids have little effect on neuropathic pain [2]. The development of effective treatments requires an understanding of the molecular and cellular mechanisms underlying neuropathic pain. Microglia, the immunocompetent cells in the central nervous system (CNS), have emerged as a pivotal player in neuropathic pain [2–6]. Microglia become activated in the spinal dorsal horn immediately after sensory nerve injury [7]. They change their shape, proliferate, and produce pro-inflammatory responses that exacerbate neuropathic pain [8, 9].

G-protein-coupled receptors (GPCRs), which are seven-transmembrane domain receptors, constitute the largest superfamily of membrane proteins. GPCRs recognize various extracellular molecules and generate intracellular signals that elicit cellular responses. GPCRs have long been a pharmacological target, and GPCR-targeting drugs currently account for ~ 34% of all drugs in the USA [10]. Given that GPCRs and their ligands play key roles in the interactions between injured neurons and neighboring glial cells [11, 12], we previously carried out a gene screen in a mouse model of motor nerve injury to identify GPCRs involved in neuron-glia interactions after nerve injury [13]. We designed primer pairs for 274 putative non-sensory GPCRs and examined changes in their expression in injured motor nuclei by quantitative real-time PCR (qRT-PCR). We successfully identified 29 candidate GPCRs whose expression levels were upregulated more than two-fold after nerve injury. Among these upregulated GPCRs, G-protein-coupled receptor 34 (GPR34) showed a substantial upregulation (~ 4-fold) after injury [13].

GPR34 was first discovered in the 1990s by two independent groups [14, 15]. Although its ligand remained unknown when we initially identified GPR34 as a candidate gene in the screening [13], lysophosphatidylserine (LysoPS), which is generated by enzymatic hydrolysis of the membrane phospholipid phosphatidylserine (PS), is currently considered a ligand for GPR34 [16, 17]. Multiple transcriptome studies have reported that GPR34 mRNA is highly enriched in microglia [18–23], suggesting its significant roles in microglial functions. A previous study investigated GPR34 function in microglia using GPR34-deficient mice [24]. The authors reported that there were no gross abnormalities in the CNS of GPR34-deficient mice [25]; however, the number and morphology of microglia were altered in certain regions of the CNS. They further found reduced phagocytic activity and increased tumor necrosis factor (TNF)-α release in GPR34-deficient microglia. In addition to the study, microglial GPR34 expression is upregulated in demyelination model induced by cuprizone [18], whereas is downregulated in epilepsy model evoked by kainic acid [26]. These observations suggest that GPR34 might regulate microglial activity in neuronal diseases. Here, we investigate GPR34 function in activated microglia using a mouse neuropathic pain model. Our findings show that a GPR34-mediated signal exacerbates neuropathic pain by promoting pro-inflammatory responses by microglia.

## Methods

### Animals

All animal procedures were conducted in accordance with the standard guidelines of the Nagoya University Graduate Schools of Medicine and the Guide for the Care and Use of Laboratory Animals. Wild-type (WT) C57BL/6 mice were purchased from Charles River Laboratories, Japan. The male mice used in our study were 8–12 weeks of age at the start of the experiments. The production of the GPR34-deficient mice on a C57BL/6 background is described in our previous study [27]. Briefly, the GPR34 gene was disrupted with a targeting vector designed to replace the GPR34 open reading frame in-frame with a LacZ-neo cassette. All mice were housed in a controlled-temperature (23 ± 1 °C) room with a 12-hr light-dark cycle (lights on from 9:00 to 21:00), and acclimated to the environment for more than 7 days before the experiments. The study was conducted with the approval of the local animal ethics committee in accordance with the regulations for animal experiments at Nagoya University (permission No. 27204, 28303, 29281, and 30178), the 3Rs (replacement, reduction, and refinement) principal of animal experiments, the Animal Protection and Management Law of Japan (105), and the Ethical Issues of the International Association for the Study of Pain [28].

### Surgical procedure for the neuropathic pain model

We used the L4 spinal nerve injury model with some modifications [9]. Briefly, after a small skin incision was made on the left side, trimming around the intervertebral foramen was performed under 3% isoflurane anesthesia. Then, the left L4 nerve was carefully dissected and cut.

### qRT-PCR

The L4 spinal cords were harvested from deeply anesthetized mice under 3% isoflurane anesthesia (*n* = 4). The spinal cords were then vertically separated along the median. Hemisections of the ipsilateral and contralateral side were horizontally divided into dorsal horn and ventral horn according to the lower margin of the substantia gelatinosa under the microscope. Dorsal horn tissues were subjected to total RNA extraction using the acid guanidine isothiocyanate/phenol/chloroform extraction method. cDNA was prepared from total RNA by reverse transcription with SuperScript III (Invitrogen). qRT-PCR was performed with StepOnePlus (Applied Biosystems) using Fast SYBR Green Master Mix (Applied Biosystems). The following primers were used: GPR34 forward: 5′-ATATGCTACAACAGCCCGGA-3′; GPR34 reverse: 5′-GAACCGAAAGGCATGGTAAG-3′; TNF-α forward: 5′-AGCCGATGGGTTGTACCTTGTCTA-3′; TNF-α reverse: 5′-TGAGATAGCAAATCGGCTGACGGT-3′; interleukin (IL)-1β forward: 5′-ACAGAATATCAACCAACAAGTGATATTCTC-3′; IL-1β reverse: 5′-GATTCTTTCCTTTGAGGCCCA-3′; IL-6 forward: 5′-ATCCAGTTGCCTTCTTGGGACTGA-3′; IL-6 reverse: 5′-TAAGCCTCCGACTTGTGAAGTGGT-3′; inducible nitric oxide synthase (iNOS) forward: 5′-GGCAGCCTGTGAGACCTTTG-3′; iNOS reverse: 5′-GAAGCGTTTCGGGATCTGAA-3′; glyceraldehyde-3-phosphate dehydrogenase (GAPDH) forward: 5′-CAAGGTCATCCCAGAGCTGA-3′; GAPDH reverse: 5′-CGGCACGTCAGATCCACGAC-3′.

The conditions for fast qRT-PCR were as follows: 1 cycle of 95 °C for 20 sec, 40 cycles of 95 °C for 3 sec, and 60 °C for 30 sec. At the end of the PCR, the samples were subjected to melting analysis to confirm amplicon specificity. Results were normalized to GAPDH. Fold-changes in gene expression were calculated using the 2^−ΔΔCt^ method.

### In situ hybridization

Mice were deeply anesthetized with a 3% isoflurane and transcardially perfused with 4% paraformaldehyde. The L4 spinal cord was dissected and postfixed in the same fixative for 15–18 hr at 4 °C. The tissue was dehydrated in 25% sucrose in 0.1 M phosphate buffer (PB) at 4 °C overnight and frozen in dry ice. Then, 7-μm-thick frozen sections were prepared using a cryostat (CM1850, Leica Biosystems). GPR34 mRNA expression was detected using the RNAscope Fluorescent Multiplex Version 2 Assay (Advanced Cell Diagnostics) according to the manufacturer’s instructions. Briefly, after sections were hybridized with RNAscope Probe-Mm-Gpr34 (No. 318201, Advanced Cell Diagnostics), signals were amplified, and color was developed with TSA Plus fluorescein (NEL741E001KT, PerkinElmer; 1:750). For in situ hybridization combined with immunohistochemistry, in situ hybridization was first performed on the sections followed by immunohistochemistry. Images were captured on a confocal microscope (FV10i, Olympus).

### Immunohistochemistry

Immunohistochemistry was performed as previously described [29, 30]. Mice were perfused with Zamboni’s fixative (0.1 M PB containing 2% paraformaldehyde and 0.2% picric acid). The L4 spinal cords were postfixed and dehydrated in 25% sucrose in 0.1 M PB and then frozen in dry ice. Floating sections were cut on a cryostat (CM1850) at 30 μm, washed in 0.01 M phosphate buffered saline (PBS), and then reacted with primary antibodies diluted in blocking solution (0.01 M PBS containing 1% bovine serum albumin, 0.1% Triton X-100 and 0.1% NaN_3_). Primary antibodies were as follows: anti-ionized calcium-binding adaptor molecule 1 (Iba1) (Abcam, RRID: AB_2224402; 1:500), anti-interferon regulatory factor 8 (IRF8) (Santa Cruz Biotechnology, RRID: AB_649510; 1:250), and anti-protein kinase C gamma (PKCγ) (#sc-211, RRID: AB_632234; Santa Cruz Biotechnology; 1:500). Signals were visualized with Alexa-488 or -594-conjugated secondary antibodies (Invitrogen). Images were taken with a confocal microscope (FV10i).

### Histological analysis

To quantify Iba1-positive cells and area, double immunofluorescence was performed using anti-Iba1 and anti-PKCγ antibodies to identify the inner lamina II. The same laser power and sensitivities on the confocal microscope (FV10i, 60x objective lens) were used to analyze the randomly chosen 30-μm-thick sections, and the areas of lamina I and outer lamina II (lamina I/IIo) were defined and calculated. Iba1-positive cells were manually counted and normalized to the area. A total of 16 sections taken from 4 animals (4 sections/animal) were examined for each time point. In these experiments, the interval of each section was at least 90 μm, and there was no possibility of repetitive counting of the same cells.

To analyze microglial surface area, volume, and process length, floating 30-μm-thick sections of the L4 spinal cord were randomly selected and stained with anti-Iba1 and anti-PKCγ antibodies. Confocal images of the entire Z-axis were acquired in a randomly chosen field of lamina I/IIo by FV10i with × 60 objective lens (1.3× zoom), and then 3D images were produced using Imaris software (version 8.1.2, Bitplane AG). For the measurement of cellular surface area and volume of microglia, surface rendering was performed based on Iba1 signals. For the quantification of process length, Imaris FilamentTracer was used. A total of 32 cells (2 cells/section, 4 sections/animal, 4 animals) were examined in WT and GPR34-deficient mice.

### Behavioral testing

The mechanical withdrawal threshold was blindly measured using 0.02–0.2 g von Frey filaments to evaluate mechanical sensitivity (*n* = 6, 8, and 4 for Figs. 5, 6, and Additional file 1: Figure S1, respectively). Mice were individually placed on the wire mesh floor, covered by an opaque chamber, and habituated to their new environment for at least 30 min before testing. Subsequently, von Frey filaments were applied to the plantar surfaces of the hindpaws. The 50% paw withdrawal threshold (PWT) was determined using the up-down method [31].

### Quantification of LysoPS by liquid chromatography–tandem mass spectrometry (LC–MS/MS)

Detection and quantification of LysoPS was performed using LC–MS/MS as described previously, with minor modification [32]. Lipids were extracted from the dorsal horn 7 days after injury (*n* = 3) using methanol (including 17:0-LPA as internal standard; final concentration of 100 nM) and stored at − 80 °C. In this study, the LC–MS/MS system consisted of an Ultimate3000 HPLC and TSQ Quantiva triple quadropole mass spectrometer (Thermo Fisher Scientific). LysoPS analyses were performed using multiple reactive monitoring (MRM) in negative mode. LC was performed using a reverse phase column (CAPCELL PAK C18 (1.5 mm I.D. × 250 mm; particle size: 3 μm)) with a gradient elution of solvent A (5 mM ammonium formate in 95% (*v*/*v*) water, pH 4.0) and solvent B (5 mM ammonium formate in 95% (*v*/*v*) acetonitrile, pH 4.0) at 200 μl/m. Gradient conditions were as follows: hold 50% B for 0.2 min, followed by a linear gradient to 100% B over 11.8 min, hold 100% B for 5 min, return to the initial condition over 0.5 min, and maintain for 2.5 min until the end of the run (total run time: 20 min).

### Intrathecal administration of the GPR34 antagonist

Intrathecal administration was performed as previously described [9]. In brief, mice were placed in a prone position under 3% isoflurane anesthesia, and an incision was made on the back. The ligaments and paraspinal muscles were partially cut, and the L5 spinous process was removed. A 32-guage catheter (ReCathCo) was intrathecally inserted to a depth of 14 mm through a dural incision made with a 22-guage needle. After nerve injury surgery, 5 μl of 10 mM GPR34 antagonist, whose IC50 is 0.3 μM determined by Ono Pharmaceutical Co, Ltd. using transforming growth factor-α shedding assay [17, 33], or saline was intrathecally injected once a day for 14 days. A 5-μl volume of saline was injected to flush the site after each antagonist injection. Behavioral testing was performed 1 hr after final administration at days 3, 7, and 14 (*n* = 8).

### Statistical analysis

Values are expressed as mean ± SEM. Data of von Frey hair test were analyzed by one-way repeated-measures (RM) ANOVA with Bonferroni adjustment or the nonparametric RM ANOVA on ranks with Dunnett’s adjustment. Other data were analyzed by one- or two-way ANOVA with post-hoc Tukey’s test. *p* < 0.05 was considered statistically significant.

## Results

### GPR34 is specifically expressed by microglia in the dorsal horn in the neuropathic pain model

**Fig. 1:**
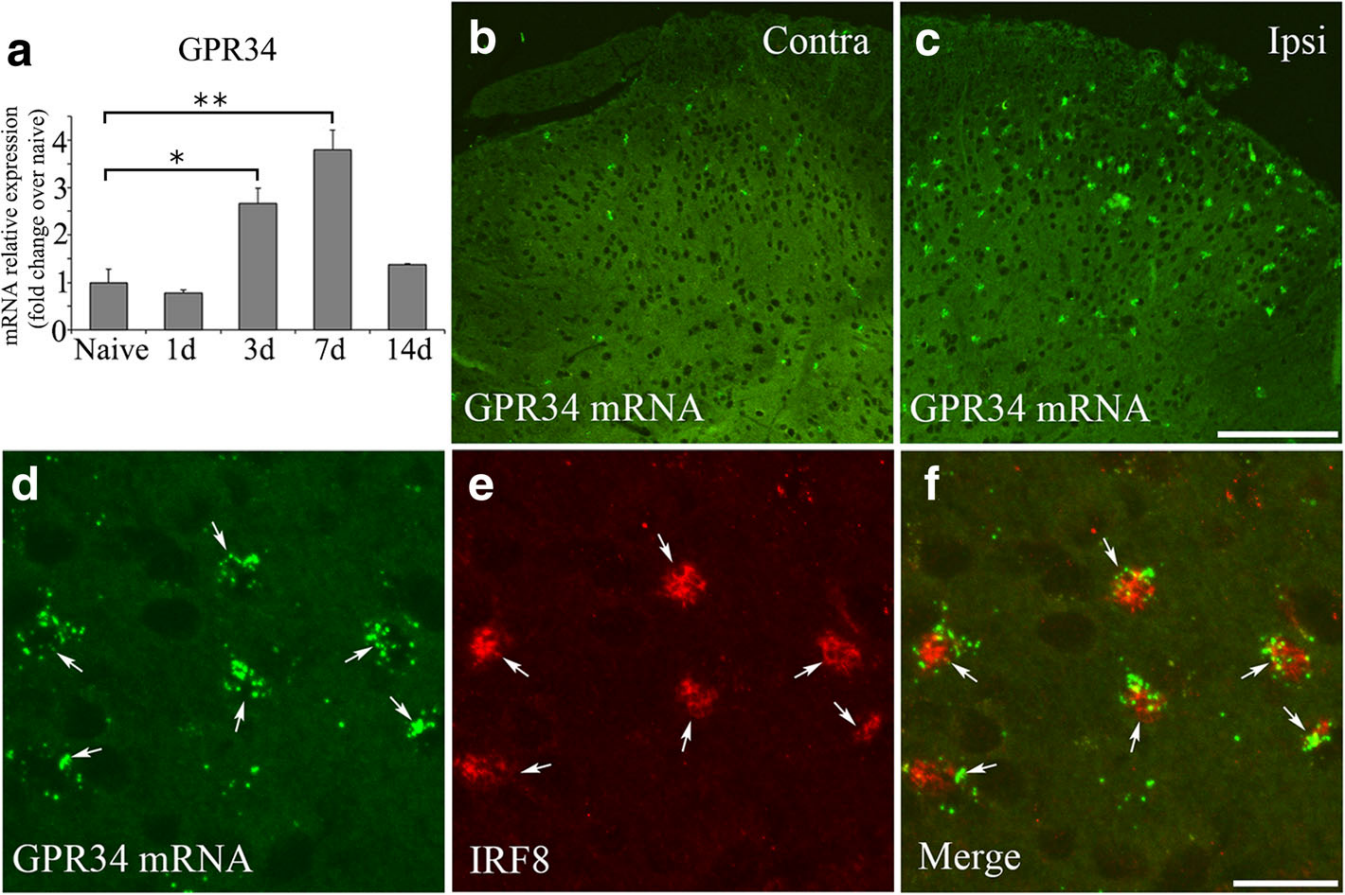
GPR34 expression is induced in microglia in the spinal dorsal horn after L4 nerve injury. **a** qRT-PCR analysis of GPR34 mRNA in the ipsilateral L4 dorsal horn of WT mice before (Naive) and after L4 nerve injury. Results are normalized to the housekeeping gene GAPDH (*n* = 4 for each time point). Values are mean ± SEM. Data are shown as fold change over naive sample. *p < 0.05, **p < 0.01 (one-way ANOVA with post hoc Tukey’s test). **b**, **c** In situ hybridization shows increased GPR34 mRNA expression in the spinal dorsal horn 7 days after L4 nerve injury (low-magnification images). *Contra* contralateral side, *Ipsi* ipsilateral side. Scale bar = 200 μm. **d**–**f** Localization of GPR34 mRNA in microglia in the ipsilateral dorsal horn (high-magnification images). Hybridized cells (**d**) display IRF8 immunoreactivity (**e**) in their nuclei (arrows). Scale bar = 30 μm

We used L4 spinal nerve transection model, which was used in our previous studies [9, 34]. Naive (non-operation) mice or contralateral side of operated mice was used as a control throughout this study, because microglial activation did not occur (Additional file 2: Figure S2) and PWT was normal (Additional file 1: Figure S1) in naive mice, sham-operated mouse, and contralateral side of operated mice. We first examined changes in GPR34 mRNA expression in spinal dorsal horn tissues at the L4 level using qRT-PCR. GPR34 mRNA was significantly increased in the ipsilateral dorsal horn from 3 days post nerve injury, with levels peaking at 7 days (Fig. 1a). To confirm that GPR34 was specifically expressed by microglia, we employed the RNAscope in situ hybridization technique, which detects mRNA with high sensitivity [35]. After in situ hybridization, we stained the sections with anti-IRF8 antibody, which was shown to stain microglial nuclei in the CNS even after in situ hybridization procedure [9, 36]. Consistent with the qRT-PCR results, the number of GPR34-positive cells was increased in the ipsilateral dorsal horn (Fig. 1b, c). Almost all the GPR34-positive cells were also IRF8-positive (Fig. 1d–f). Because circulating monocytes do not infiltrate spinal cord in our model [9], these results indicate that GPR34 is predominantly expressed by microglia in the dorsal horn after sensory nerve injury.

### Microglial numbers and morphology appear to be unaffected by the absence of GPR34

**Fig. 2:**
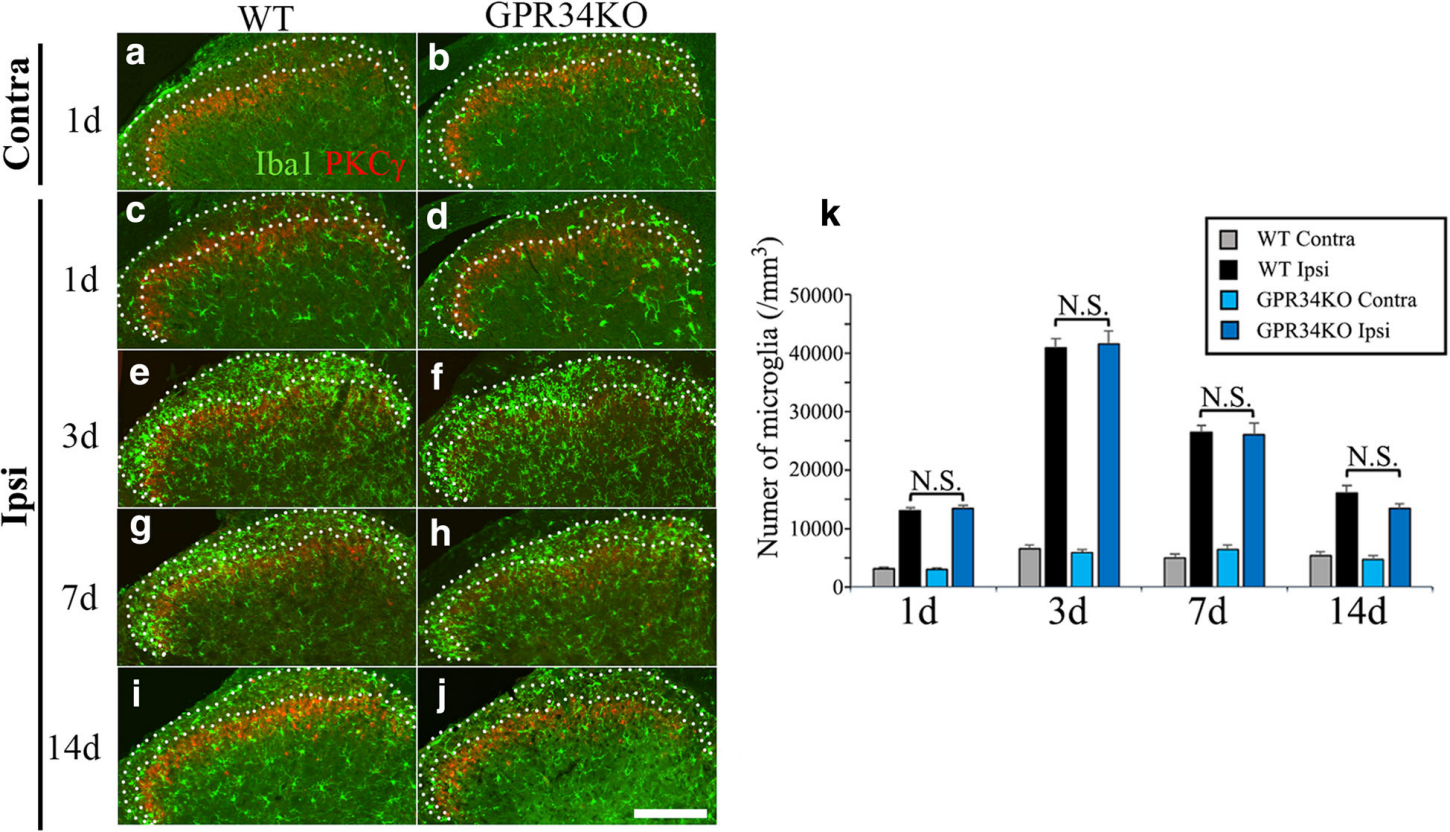
Microglial numbers are not affected by GPR34 deficiency. **a**, **b** Contralateral dorsal horn in WT (**a**) and GPR34-deficient (**b**) mice at 1 day after L4 nerve injury. **c**–**j** Ipsilateral dorsal horn in WT (**c**, **e**, **g**, **i**) and GPR34-deficient (**d**, **f**, **h**, **j**) mice at 1 (**c**, **d**), 3 (**e**, **f**), 7 (**g**, **h**), and 14 (**i**, **j**) days after L4 nerve injury. Sections were stained by anti-Iba1 antibody (green) and anti-PKCγ antibody (red) for visualization of microglia and inner lamina II, respectively. Iba1-positive microglia within lamina I/IIo, identified by a dotted line, were counted. Scale bar = 200 μm. **k** Quantification of microglial numbers in lamina I/IIo of the ipsilateral and contralateral L4 dorsal horn in WT and GPR34-deficient mice after L4 nerve injury (*n* = 4 for each time point). Values are mean ± SEM. There was no significant difference between WT and GPR34-deficient mice at any time points (one-way ANOVA with post-hoc Tukey’s test)

Activated microglia proliferate in the dorsal horn after sensory nerve injury [7]. Thus, microglial numbers were evaluated by Iba1 staining to identify the difference between WT and GPR34-deficient mice, in which GPR34 mRNA was not detected in a confirmative experiment (Additional file 3: Figure S3). We quantified Iba1-positive microglial numbers in lamina I/IIo, which are pivotal layers of the spinal cord in the pathogenesis of neuropathic pain (Fig. 2a–j). To identify lamina I/IIo, an antibody against PKCγ was used to label inner lamina II [37]. Microglial numbers were increased from 1 day post injury in ipsilateral lamina I/IIo. However, there was no significant difference between WT and GPR34-deficient mice at any time point (Fig. 2k). These results suggest that a GPR34-mediated signal is not involved in microglial proliferation or migration after nerve injury.

**Fig. 3:**
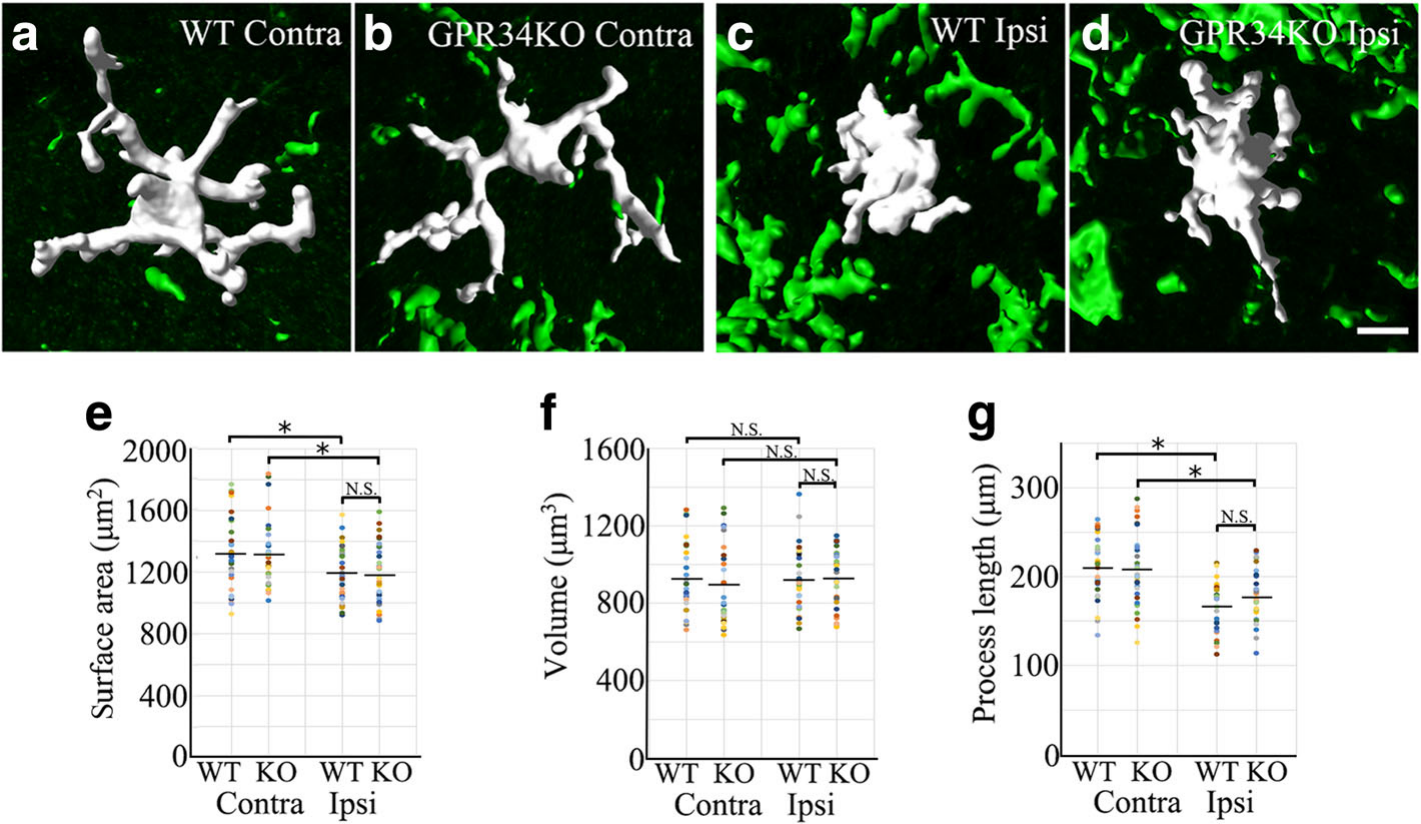
Microglial morphology is not affected by GPR34 deficiency. **a**–**d** Representative 3D reconstructions of microglia in the contralateral (Contra) and ipsilateral (Ipsi) dorsal horn in WT and GPR34-deficient mice. Scale bar = 10 μm. **e**–**g** Quantitative morphometric analysis of microglial surface area (**e**), volume (**f**), and process length (**g**) (*n* = 32 cells from four animals). Values are mean ± SEM. *p < 0.05 (one-way ANOVA with post-hoc Tukey’s test). There was no significant difference in any parameter between WT and GPR34-deficient mice

Although microglia exhibit a ramified shape in the quiescent state, they undergo a dramatic morphological change in the dorsal horn in response to sensory nerve injury [7]. To evaluate microglial morphology, a morphometric analysis was performed. The 3D morphology of microglia was reconstructed from confocal images using Imaris software, and we analyzed microglial surface area, volume, and process length (Fig. 3). There were no significant differences in any of the three parameters between WT and GPR34-deficient mice, suggesting that a GPR34-mediated signal does not contribute to microglial morphological changes after injury.

### GPR34 deficiency reduces pro-inflammatory responses in microglia

**Fig. 4:**
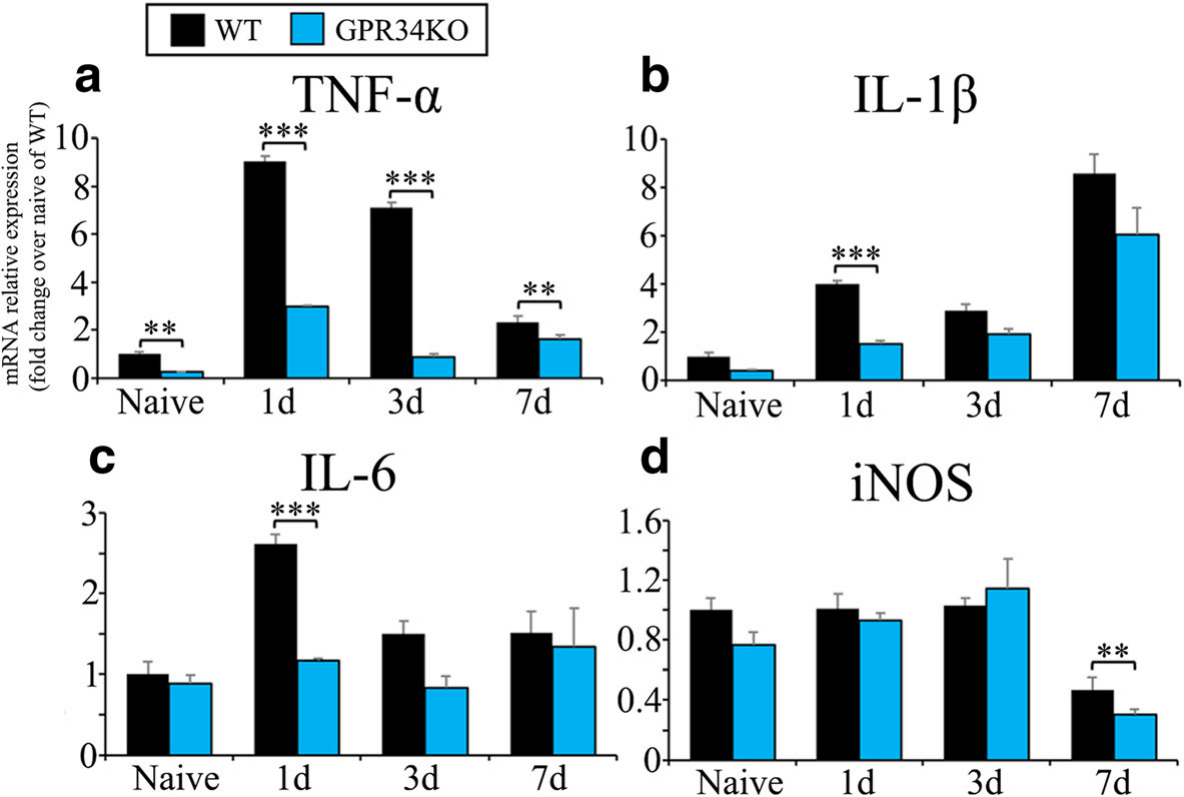
Pro-inflammatory microglial molecules are reduced in GPR34-deficient mice. The ipsilateral L4 dorsal horn was obtained from WT and GPR34-deficient mice before (Naive) and after L4 nerve injury (*n* = 4 for each time point), and mRNA expression levels of neuropathic pain-related molecules were quantified by qRT-PCR. **a** TNF-α, **b** IL-1β, **c** IL-6, and **d** iNOS. Results are normalized to GAPDH. Data are shown as fold change over naive sample of WT mice. Values are mean ± SEM. **p < 0.01, ***p < 0.001 (two-way ANOVA with post-hoc Tukey’s test)

Activated microglia elicit neuroinflammation in the dorsal horn after sensory nerve injury, and the pro-inflammatory cytokines produced by these cells, such as TNF-α, are associated with neuropathic pain [8, 38]. Thus, we analyzed the expression of pro-inflammatory cytokines by qRT-PCR in the ipsilateral dorsal horn after L4 spinal nerve injury. At 1 day after injury, mRNA levels of TNF-α, IL-1β, and IL-6 were increased in the dorsal horn; however, they were significantly lower in GPR34-deficient mice compared with WT mice (Fig. 4a–c). TNF-α and IL-1β mRNA levels were overall downregulated after nerve injury in GPR34-deficient mice. Expression of iNOS mRNA was significantly lower in GPR34-deficient mice at 7 days, but was similar until 3 days (Fig. 4a). Because microglial numbers in the dorsal horn were similar in WT and GPR34-deficient mice (Fig. 2), the reductions in the expression of pro-inflammatory molecules in GPR34-deficient mice were likely caused by suppressed expression of pro-inflammatory cytokines in microglia rather than a decrease in microglial numbers. These findings therefore suggest that a GPR34-mediated signal elicits pro-inflammatory responses in microglia activated by injury.

### GPR34 deficiency reduces neuropathic pain induced by sensory nerve injury

**Fig. 5:**
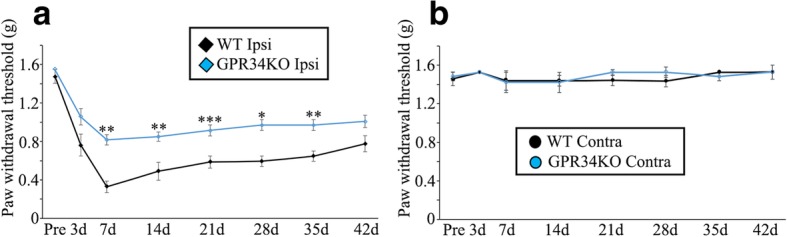
Nerve injury-induced mechanical allodynia is reduced in GPR34-deficient mice. PWT in WT and GPR34-deficient mice before (pre) and after L4 nerve injury (*n* = 6). PWT in the ipsilateral (Ipsi) (**a**) and contralateral (Contra) (**b**) side. *p < 0.05, **p < 0.01, ***p < 0.001 (one-way RM ANOVA with Bonferroni adjustment or the nonparametric RM ANOVA on ranks with Dunnett’s adjustment). On the ipsilateral side, the PWT was higher in GPR34-deficient mice than in WT mice from 7 to 35 days

Because microglial pro-inflammatory responses affect the development and persistence of neuropathic pain [2, 39], we analyzed the impact of GPR34 deficiency on mechanical allodynia. We measured the PWT to von Frey filament stimulation of the hindpaw in WT and GPR34-deficient mice following unilateral L4 nerve injury. There was a decrease in the PWT from 3 to 42 days post nerve injury in the ipsilateral side of WT mice, and GPR34-deficient mice showed significantly higher PWTs than WT mice from 7 to 35 days post injury (Fig. 5a). There was no significant difference in the contralateral PWT between WT and GPR34-deficient mice at any of the time points (Fig. 5b). These results suggest that a GPR34-mediated signal in microglia exacerbates neuropathic pain. However, since GPR34 has been reported to be expressed in other immune cells than microglia, such as monocytes and dendritic cells [25, 40, 41], which infiltrate injured nerve and evoke inflammation [42, 43], GPR34 deficiency in those infiltrated immune cells may also contribute to the higher PWT in GPR34-deficient mice.

### Inhibition of LysoPS-GPR34 signaling suppresses neuropathic pain

**Fig. 6:**
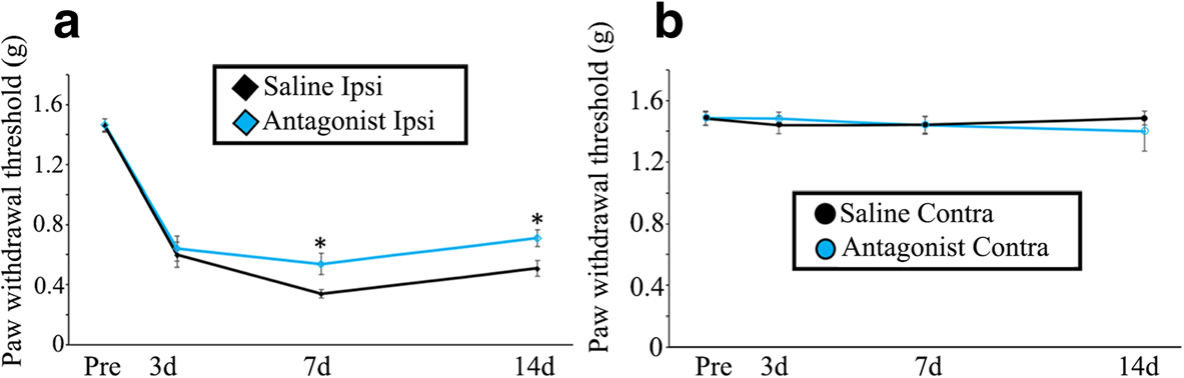
Neuropathic pain is reduced by GPR34 antagonism. The PWT in the ipsilateral (Ipsi) (**a**) and contralateral (Contra) (**b**) side of WT mice with L4 nerve injury. Mice were intrathecally administered the GPR34 antagonist once a day for 14 days after nerve injury (*n* = 8), and the von Frey test was performed on days 3, 7, and 14. Values are mean ± SEM. *p < 0.05 (one-way RM ANOVA with Bonferroni adjustment or the nonparametric RM ANOVA on ranks with Dunnett’s adjustment)

We evaluated the concentration of the GPR34 ligand, LysoPS, in the dorsal horn after nerve injury (Additional file 4: Figure S4). LC–MS/MS analysis of representative LysoPS species demonstrated that the amount of 22:6-LysoPS was almost unchanged. The level of 18:0-LysoPS and 18:1-LysoPS tended to increase (18:0-LysoPS: *p* = 0.094; 18:1-LysoPS: *p* = 0.088), although there were no statistical significances in the increment. Nevertheless, the existence of significant amount of LysoPS species became obvious. Therefore, we expected that LysoPS-GPR34 signaling pathway was activated in the injured dorsal horn, and examined the effect of a GPR34 antagonist. We evaluated pain behavior in WT mice intrathecally administrated the GPR34 antagonist once a day for 14 days (Fig. 6). At 7 and 14 days, pain behaviors were improved in antagonist-treated mice, suggesting that LysoPS stimulates GPR34 to enhance neuropathic pain.

## Discussion

Although GPR34 is known to be expressed by microglia [18], its role remained unclear, especially in neural diseases. In this study, we used a mouse neuropathic pain model to examine its function in microglia. We found that GPR34 is specifically expressed by microglia in the dorsal horn, and that its expression is increased in response to nerve injury (Fig. 1). Concomitantly, its ligand, LysoPS, was significantly detected in the dorsal horn (Additional file 4: Figure S4), suggesting that the GPR34 signaling pathway is activated in microglia following nerve injury and participates in regulating microglial functions. Indeed, GPR34 deficiency reduced expression of pro-inflammatory cytokines, which are assumed to be released from microglia [44], in the dorsal horn after nerve injury (Fig. 4), although GPR34 signaling did not affect the number or morphology of microglia (Figs. 2 and 3). Concurrently, the GPR34 deficiency resulted in a decrease in mechanical allodynia (Fig. 5). In addition, intrathecal administration of the GPR34 antagonist significantly suppressed pain behavior in injured mice (Fig. 6). These findings suggest that LysoPS induces microglial pro-inflammatory responses via GPR34, resulting in the development of neuropathic pain after sensory nerve injury.

Microglia are activated in response to various stimuli such as traumatic injury and inflammation, and exert various effects on surrounding cells [45]. Receptors expressed on the microglial cell surface are thought to play critical roles in their activation. Several GPCRs are expressed predominantly by microglia, and have been extensively studied. For example, the fractalkine receptor, CX3C chemokine receptor 1 (CX3CR1), regulates microglial activity in neuronal disorders, and its deficiency significantly affects neuronal fate [46]. Furthermore, some purinergic GPCRs, such as P2X4, P2X7, and P2Y12, are essential for microglial activation and pain exacerbation in the spinal dorsal horn after sensory nerve injury [47–49]. In addition to these GPCRs, our previous study, in which we screened for GPCRs upregulated in the injured motor nucleus, identified a number of microglial-specific GPCRs [13], including CC chemokine receptor 5 (CCR5) and GPR84, which were demonstrated as regulators of microglial pro-inflammatory responses and motility, respectively [13, 50]. Intriguingly, we also identified several receptors for lipid mediators, including GPR84, a receptor for medium-chain fatty acids; GPR34, a receptor for LysoPS; and EDG5, a receptor for sphingosine-1-phosphate [13]. These observations suggest that several lipid mediator signaling pathways participate in the regulation of microglial activity after nerve injury.

When we initially identified GPR34 in the screen for upregulated GPCRs [13], the receptor was an orphan. Recently, LysoPS has been identified as the ligand for GPR34 [16, 17, 33]. GPR34 is revealed to be highly expressed by microglia [18–23]; however, most studies demonstrated microglial GPR34 expression solely by transcriptome analysis of acutely isolated microglia from mouse brain, and there has been only one histological study of GPR34 expression in the CNS [18]. Our study employing high sensitivity in situ hybridization combined with immunohistochemistry showed that GPR34 mRNA is predominantly expressed by microglia (Fig. 1d–f). After sensory nerve injury, microglial numbers peaked 3 days post injury, whereas GPR34 mRNA levels peaked 7 days post injury in the dorsal horn (Figs. 1a and 2). This suggests that the induction of GPR34 expression is preceded by the proliferation and activation of microglia, which are triggered by other signaling pathways (e.g., purinergic signaling). This might explain why GPR34 deficiency does not affect cell number or morphology (Figs. 2 and 3).

A previous study proposed that GPR34 is an anti-inflammatory receptor [24]. The authors showed enhanced expression of IL-1β mRNA in acutely isolated GPR34-deficient microglia as well as higher levels of TNF-α release in GPR34-deficient cultured microglia in vitro [24]. In contrast, our present findings suggest that GPR34 is a pro-inflammatory receptor that induces the expression of microglial pro-inflammatory molecules, such as TNF-α, IL-1β, IL-6, and iNOS, in the dorsal horn after injury in vivo (Fig. 4). Two lines of evidence support our current results. First, in GPR34-deficient mice, our behavioral test clearly showed attenuated pain behavior (Fig. 5), which was reflected by suppressed pro-inflammatory responses in microglia [51]. Second, in other cell types, a GPR34-mediated signal activates Akt, extracellular signal-regulated kinase (ERK), and nuclear factor (NF)-κB [41, 52, 53], which are all known inducers of pro-inflammatory molecules in microglia [54–57]. Therefore, at least in the neuropathic pain model, a GPR34-mediated signal stimulates pro-inflammatory responses in microglia.

Previous reports, including those from our group, demonstrated LysoPS to be an endogenous ligand for GPR34 [16, 17, 33], although controversy remains [58–60]. The hydrolysis of membrane PS results in the generation and release of LysoPS into the extracellular space, where it functions as an intercellular signal that regulates immune cell functions, such as phagocytosis in macrophages [61], proliferation of T cells [62], and degranulation of mast cells [63]. Here, we found that the dorsal horn does contain significant amount of LysoPS species (Additional file 4: Figure S4). Although the functions of LysoPS in the CNS in vivo remain unclear, it likely regulates microglial activity. Indeed, homozygous mutation of the gene for α/β-hydrolase domain-containing 12 (ABHD12), which hydrolyzes LysoPS, causes a neurological disorder, termed polyneuropathy, hearing loss, ataxia, retinosis pigmentosa, and cataract (PHARC) [64]. In ABHD12-deficient mice, accumulated LysoPS enhances microglial activation in the brain to cause PHARC-like defects [65]. Our current results are in line with these observations (Figs. 4, 5, and 6). The pain suppression effect of GPR34-deficient mice and GPR34 antagonist was statistically significant, however not drastic (Fig. 6), which may be due to the fact that GPR34 is one of the activation receptors expressed in microglia [51]. It is also possible that LysoPS may activate spinal microglia via receptors other than GPR34, since saturated fatty acids was shown to activate toll-like receptors to induce inflammation in macrophages [66].

Our recent in vitro study revealed that LysoPS induces morphological changes in primary cultured microglia (i.e., from an amoeboid to a ramified shape) in a GPR34-independent manner [27]. LysoPS added to the culture media can easily incorporate into the microglial plasma membrane and recruit Cdc42 to remodel the actin cytoskeleton. As this morphological change is observed in GPR34-deficient microglia, it most likely occurs in a receptor independent manner. This phenomenon is only observed in cultured microglia [27]. Therefore, the mechanisms underlying the induction of pro-inflammatory molecules and pain behavior in our present in vivo study appear to be distinct from those involved in vitro.

## Conclusions

We found that LysoPS signals act on microglial GPR34 to elicit pro-inflammatory responses in activated microglia. In addition to traumatic injury, microglial-mediated neuroinflammation also occurs in neurodegenerative diseases, where it affects neuronal function and survival, either beneficially or detrimentally [67, 68]. Therefore, GPR34 is a potential therapeutic target for neurodegenerative diseases and neural injury. However, further studies are needed to identify the cellular source of LysoPS and to provide further mechanistic insight into the role of LysoPS and GPR34 in the CNS.

## Additional files

Additional file 1: **Figure S2.** Pain sensitivity is normal in control groups. PWT was measured in non-operated mice (Naive), sham-operated mice (Sham), and L4 nerve operated mice 0 (Pre), 3 and 7 days after injury (*n* = 4). ***p<0.001 (one-way RM ANOVA with Bonferroni adjustment or the nonparametric RM ANOVA on ranks with Dunnett’s adjustment). PWT of sham-operated mice and the contralateral paw of operated mice (Contra) was equivalent to that of naive mice. In contrast, PWT was significantly decreased in the ipsilateral side of operated mice (Ipsi). (DOCX 48 kb)

Additional file 2: **Figure S1.** Microglial activation does not occur in control groups. Dorsal horn tissues at the L4 level were obtained 7 days after injury (*n* = 4), and mRNA expression of a microglial marker, Iba1 (a), and a prominently upregulated cytokine at day 7, IL-1β (b), was analyzed by qRT-PCR. Results are normalized to GAPDH and shown as fold change over naive sample. Expression of both molecules in sham-operated mice (Sham) and the contralateral side of operated mice (Contra) was equivalent to that in non-operated mice (Naive). In contrast, expression of both molecules was significantly increased in the ipsilateral side of operated mice (Ipsi). Values are mean ± SEM. **p < 0.01, ***p < 0.001 (one-way ANOVA with post hoc Turkey’s test). (DOCX 51 kb)

Additional file 3: **Figure S3.** GPR34 mRNA is not detected in GPR34-deficient mice. Dorsal horn tissues at the L4 level were obtained from non-operated WT and GPR34-deficient mice (*n* = 4), and mRNA expression of GPR34 was analyzed by qRT-PCR. Results are normalized to GAPDH. Data are shown as fold change over WT sample. Values are mean ± SEM. ***p<0.001 (one-way ANOVA with post hoc Turkey’s test). (DOCX 40 kb)

Additional file 4: **Figure S4.** The dorsal horn contains LysoPS species. Quantification of representative LysoPS species (18:0-LysoPS, 18:1-LysoPS, 22:6-LysoPS) in the contralateral (Contra) and ipsilateral (Ipsi) dorsal horn 7 days after injury, using LC–MS/MS (*n* = 3). Contralateral side was used as a control. Amounts of 18:0-LysoPS and 18:1-LysoPS tended to increase in the injured dorsal horn, although the increases were not significant (one-way ANOVA with post hoc Tukey’s test). (DOCX 67 kb)

## Abbreviations

ABHD12: α/β-Hydrolase domain-containing 12
CCR5: CC chemokine receptor 5
CNS: Central nervous system
CX3CR1: CX3C chemokine receptor 1
ERK: Extracellular signal-regulated kinase
GAPDH: Glyceraldehyde-3-phosphate dehydrogenase
GPCR: G-protein-coupled receptor
GPR34: G-protein-coupled receptor 34
Iba1: Ionized calcium-binding adaptor molecule 1
IL: Interleukin
iNOS: Inducible nitric oxide synthase
IRF8: Interferon regulatory factor 8
Lamina I/IIo: Lamina I and outer lamina II
LC–MS/MS: Liquid chromatography–tandem mass spectrometry
LysoPS: Lysophosphatidylserine
MRM: Multiple reactive monitoring
NF: Nuclear factor
PB: Phosphate buffer
PBS: Phosphate-buffered saline
PHARC: Polyneuropathy, hearing loss, ataxia, retinosis pigmentosa, and cataract
PKCγ: Protein kinase C gamma
PS: Phosphatidylserine
PWT: Paw withdrawal threshold
qRT-PCR: Quantitative real-time PCR
RM: repeated-measures
TNF: Tumor necrosis factor
WT: Wild-type

## References

[1] Inoue K (2006). ATP receptors of microglia involved in pain. Novartis Found Symp.

[2] Inoue K, Tsuda M (2009). Microglia and neuropathic pain. Glia..

[3] Yasui M, Yoshimura T, Takeuchi S, Tokizane K, Tsuda M, Inoue K, Kiyama H (2014). A chronic fatigue syndrome model demonstrates mechanical allodynia and muscular hyperalgesia via spinal microglial activation. Glia..

[4] Grace PM, Hutchinson MR, Maier SF, Watkins LR (2014). Pathological pain and the neuroimmune interface. Nat Rev Immunol.

[5] Maeda M, Tsuda M, Tozaki-Saitoh H, Inoue K, Kiyama H (2010). Nerve injury-activated microglia engulf myelinated axons in a P2Y12 signaling-dependent manner in the dorsal horn. Glia..

[6] Coyle D (1998). Partial peripheral nerve injury leads to activation of astroglia and microglia which parallels the development of allodynic behavior. Glia..

[7] Kohno K, Kitano J, Kohro Y, Tozaki-Saitoh H, Inoue K, Tsuda M (2018). Temporal kinetics of microgliosis in the spinal dorsal horn after peripheral nerve injury in rodents. Biol Pharm Bull.

[8] Leung L, Cahill CM (2010). TNF-alpha and neuropathic pain-a review. J Neuroinflammation.

[9] Kobayashi M, Konishi H, Sayo A, Takai T, Kiyama H (2016). TREM2/DAP12 signal elicits Proinflammatory response in microglia and exacerbates neuropathic pain. J Neurosci.

[10] Hauser AS, Attwood MM, Rask-Andersen M, Schioth HB, Gloriam DE (2017). Trends in GPCR drug discovery: new agents, targets and indications. Nat Rev Drug Discov.

[11] Fields RD, Burnstock G (2006). Purinergic signalling in neuron-glia interactions. Nat Rev Neurosci.

[12] Old EA, Malcangio M (2012). Chemokine mediated neuron-glia communication and aberrant signalling in neuropathic pain states. Curr Opin Pharmacol.

[13] Gamo K, Kiryu-Seo S, Konishi H, Aoki S, Matsushima K, Wada K, Kiyama H (2008). G-protein-coupled receptor screen reveals a role for chemokine receptor CCR5 in suppressing microglial neurotoxicity. J Neurosci.

[14] Marchese A, Sawzdargo M, Nguyen T, Cheng R, Heng HH, Nowak T, Im DS, Lynch KR, George SR, O'Dowd BF (1999). Discovery of three novel orphan G-protein-coupled receptors. Genomics..

[15] Schoneberg T, Schulz A, Grosse R, Schade R, Henklein P, Schultz G, Gudermann T (1999). A novel subgroup of class I G-protein-coupled receptors. Biochim Biophys Acta.

[16] Sugo T, Tachimoto H, Chikatsu T, Murakami Y, Kikukawa Y, Sato S, Kikuchi K, Nagi T, Harada M, Ogi K (2006). Identification of a lysophosphatidylserine receptor on mast cells. Biochem Biophys Res Commun.

[17] Inoue A, Ishiguro J, Kitamura H, Arima N, Okutani M, Shuto A, Higashiyama S, Ohwada T, Arai H, Makide K, Aoki J (2012). TGFalpha shedding assay: an accurate and versatile method for detecting GPCR activation. Nat Methods.

[18] Bedard A, Tremblay P, Chernomoretz A, Vallieres L (2007). Identification of genes preferentially expressed by microglia and upregulated during cuprizone-induced inflammation. Glia..

[19] Hickman SE, Kingery ND, Ohsumi TK, Borowsky ML, Wang LC, Means TK, El Khoury J (2013). The microglial sensome revealed by direct RNA sequencing. Nat Neurosci.

[20] Butovsky O, Jedrychowski MP, Moore CS, Cialic R, Lanser AJ, Gabriely G, Koeglsperger T, Dake B, Wu PM, Doykan CE (2014). Identification of a unique TGF-beta-dependent molecular and functional signature in microglia. Nat Neurosci.

[21] Bennett ML, Bennett FC, Liddelow SA, Ajami B, Zamanian JL, Fernhoff NB, Mulinyawe SB, Bohlen CJ, Adil A, Tucker A (2016). New tools for studying microglia in the mouse and human CNS. Proc Natl Acad Sci U S A.

[22] Artegiani B, Lyubimova A, Muraro M, van Es JH, van Oudenaarden A, Clevers H (2017). A single-cell RNA sequencing study reveals cellular and molecular dynamics of the hippocampal neurogenic niche. Cell Rep.

[23] Tischner D, Grimm M, Kaur H, Staudenraus D, Carvalho J, Looso M, Gunther S, Wanke F, Moos S, Siller N, et al. Single-cell profiling reveals GPCR heterogeneity and functional patterning during neuroinflammation. JCI Insight. 2017;2:e95063. 10.1172/jci.insight.95063 PMC5543912 28768912

[24] Preissler J, Grosche A, Lede V, Le Duc D, Krugel K, Matyash V, Szulzewsky F, Kallendrusch S, Immig K, Kettenmann H (2015). Altered microglial phagocytosis in GPR34-deficient mice. Glia..

[25] Schoneberg T, Meister J, Knierim AB, Schulz A (2018). The G protein-coupled receptor GPR34—the past 20years of a grownup. Pharmacol Ther.

[26] Abiega O, Beccari S, Diaz-Aparicio I, Nadjar A, Laye S, Leyrolle Q, Gomez-Nicola D, Domercq M, Perez-Samartin A, Sanchez-Zafra V (2016). Neuronal hyperactivity disturbs ATP microgradients, impairs microglial motility, and reduces phagocytic receptor expression triggering apoptosis/microglial phagocytosis uncoupling. PLoS Biol.

[27] Tokizane K, Konishi H, Makide K, Kawana H, Nakamuta S, Kaibuchi K, Ohwada T, Aoki J, Kiyama H (2017). Phospholipid localization implies microglial morphology and function via Cdc42 in vitro. Glia..

[28] Zimmermann M (1983). Ethical guidelines for investigations of experimental pain in conscious animals. Pain..

[29] Konishi H, Namikawa K, Kiyama H (2006). Annexin III implicated in the microglial response to motor nerve injury. Glia..

[30] Kobayashi M, Konishi H, Takai T, Kiyama H (2015). A DAP12-dependent signal promotes pro-inflammatory polarization in microglia following nerve injury and exacerbates degeneration of injured neurons. Glia..

[31] Chaplan SR, Bach FW, Pogrel JW, Chung JM, Yaksh TL (1994). Quantitative assessment of tactile allodynia in the rat paw. J Neurosci Methods.

[32] Okudaira M, Inoue A, Shuto A, Nakanaga K, Kano K, Makide K, Saigusa D, Tomioka Y, Aoki J (2014). Separation and quantification of 2-acyl-1-lysophospholipids and 1-acyl-2-lysophospholipids in biological samples by LC-MS/MS. J Lipid Res.

[33] Kitamura H, Makide K, Shuto A, Ikubo M, Inoue A, Suzuki K, Sato Y, Nakamura S, Otani Y, Ohwada T, Aoki J (2012). GPR34 is a receptor for lysophosphatidylserine with a fatty acid at the sn-2 position. J Biochem.

[34] Konishi H, Kobayashi M, Kunisawa T, Imai K, Sayo A, Malissen B, Crocker PR, Sato K, Kiyama H (2017). Siglec-H is a microglia-specific marker that discriminates microglia from CNS-associated macrophages and CNS-infiltrating monocytes. Glia..

[35] Wang F, Flanagan J, Su N, Wang LC, Bui S, Nielson A, Wu X, Vo HT, Ma XJ, Luo Y (2012). RNAscope: a novel in situ RNA analysis platform for formalin-fixed, paraffin-embedded tissues. J Mol Diagn.

[36] Masuda T, Tsuda M, Yoshinaga R, Tozaki-Saitoh H, Ozato K, Tamura T, Inoue K (2012). IRF8 is a critical transcription factor for transforming microglia into a reactive phenotype. Cell Rep.

[37] Malmberg AB, Chen C, Tonegawa S, Basbaum AI (1997). Preserved acute pain and reduced neuropathic pain in mice lacking PKCgamma. Science..

[38] He XH, Zang Y, Chen X, Pang RP, Xu JT, Zhou X, Wei XH, Li YY, Xin WJ, Qin ZH, Liu XG (2010). TNF-alpha contributes to up-regulation of Nav1.3 and Nav1.8 in DRG neurons following motor fiber injury. Pain..

[39] Tsuda M, Masuda T, Tozaki-Saitoh H, Inoue K (2013). P2X4 receptors and neuropathic pain. Front Cell Neurosci.

[40] Papatheodorou I, Fonseca NA, Keays M, Tang YA, Barrera E, Bazant W, Burke M, Fullgrabe A, Fuentes AM, George N (2018). Expression Atlas: gene and protein expression across multiple studies and organisms. Nucleic Acids Res.

[41] Jager E, Schulz A, Lede V, Lin CC, Schoneberg T, Le Duc D (2016). Dendritic cells regulate GPR34 through Mitogenic signals and undergo apoptosis in its absence. J Immunol.

[42] Beuche W, Friede RL (1984). The role of non-resident cells in Wallerian degeneration. J Neurocytol.

[43] Kim CF, Moalem-Taylor G (2011). Interleukin-17 contributes to neuroinflammation and neuropathic pain following peripheral nerve injury in mice. J Pain.

[44] Inoue K (2006). The function of microglia through purinergic receptors: neuropathic pain and cytokine release. Pharmacol Ther.

[45] Kettenmann H, Hanisch UK, Noda M, Verkhratsky A (2011). Physiology of microglia. Physiol Rev.

[46] Cardona AE, Pioro EP, Sasse ME, Kostenko V, Cardona SM, Dijkstra IM, Huang D, Kidd G, Dombrowski S, Dutta R (2006). Control of microglial neurotoxicity by the fractalkine receptor. Nat Neurosci.

[47] Tsuda M, Shigemoto-Mogami Y, Koizumi S, Mizokoshi A, Kohsaka S, Salter MW, Inoue K (2003). P2X4 receptors induced in spinal microglia gate tactile allodynia after nerve injury. Nature..

[48] Tozaki-Saitoh H, Tsuda M, Miyata H, Ueda K, Kohsaka S, Inoue K (2008). P2Y12 receptors in spinal microglia are required for neuropathic pain after peripheral nerve injury. J Neurosci.

[49] He WJ, Cui J, Du L, Zhao YD, Burnstock G, Zhou HD, Ruan HZ (2012). Spinal P2X(7) receptor mediates microglia activation-induced neuropathic pain in the sciatic nerve injury rat model. Behav Brain Res.

[50] Wei L, Tokizane K, Konishi H, Yu HR, Kiyama H (2017). Agonists for G-protein-coupled receptor 84 (GPR84) alter cellular morphology and motility but do not induce pro-inflammatory responses in microglia. J Neuroinflammation.

[51] Inoue K, Tsuda M (2018). Microglia in neuropathic pain: cellular and molecular mechanisms and therapeutic potential. Nat Rev Neurosci.

[52] Ansell SM, Akasaka T, McPhail E, Manske M, Braggio E, Price-Troska T, Ziesmer S, Secreto F, Fonseca R, Gupta M (2012). t(X;14)(p11;q32) in MALT lymphoma involving GPR34 reveals a role for GPR34 in tumor cell growth. Blood..

[53] Zuo B, Li M, Liu Y, Li K, Ma S, Cui M, Qin Y, Zhu H, Pan X, Guo J (2015). G-protein coupled receptor 34 activates Erk and phosphatidylinositol 3-kinase/Akt pathways and functions as alternative pathway to mediate p185Bcr-Abl-induced transformation and leukemogenesis. Leuk Lymphoma.

[54] Heyen JR, Ye S, Finck BN, Johnson RW (2000). Interleukin (IL)-10 inhibits IL-6 production in microglia by preventing activation of NF-kappaB. Brain Res Mol Brain Res.

[55] Hide I, Tanaka M, Inoue A, Nakajima K, Kohsaka S, Inoue K, Nakata Y (2000). Extracellular ATP triggers tumor necrosis factor-alpha release from rat microglia. J Neurochem.

[56] Wang W, Ji P, Dow KE (2003). Corticotropin-releasing hormone induces proliferation and TNF-alpha release in cultured rat microglia via MAP kinase signalling pathways. J Neurochem.

[57] Saponaro C, Cianciulli A, Calvello R, Dragone T, Iacobazzi F, Panaro MA (2012). The PI3K/Akt pathway is required for LPS activation of microglial cells. Immunopharmacol Immunotoxicol.

[58] Yin H, Chu A, Li W, Wang B, Shelton F, Otero F, Nguyen DG, Caldwell JS, Chen YA (2009). Lipid G protein-coupled receptor ligand identification using beta-arrestin PathHunter assay. J Biol Chem.

[59] Ritscher L, Engemaier E, Staubert C, Liebscher I, Schmidt P, Hermsdorf T, Rompler H, Schulz A, Schoneberg T (2012). The ligand specificity of the G-protein-coupled receptor GPR34. Biochem J.

[60] Southern C, Cook JM, Neetoo-Isseljee Z, Taylor DL, Kettleborough CA, Merritt A, Bassoni DL, Raab WJ, Quinn E, Wehrman TS (2013). Screening beta-arrestin recruitment for the identification of natural ligands for orphan G-protein-coupled receptors. J Biomol Screen.

[61] Frasch SC, Bratton DL (2012). Emerging roles for lysophosphatidylserine in resolution of inflammation. Prog Lipid Res.

[62] Bellini F, Bruni A (1993). Role of a serum phospholipase A1 in the phosphatidylserine-induced T cell inhibition. FEBS Lett.

[63] Martin TW, Lagunoff D (1979). Interactions of lysophospholipids and mast cells. Nature..

[64] Fiskerstrand T, H’Mida-Ben Brahim D, Johansson S, M’Zahem A, Haukanes BI, Drouot N, Zimmermann J, Cole AJ, Vedeler C, Bredrup C (2010). Mutations in ABHD12 cause the neurodegenerative disease PHARC: an inborn error of endocannabinoid metabolism. Am J Hum Genet.

[65] Blankman JL, Long JZ, Trauger SA, Siuzdak G, Cravatt BF (2013). ABHD12 controls brain lysophosphatidylserine pathways that are deregulated in a murine model of the neurodegenerative disease PHARC. Proc Natl Acad Sci U S A.

[66] Huang S, Rutkowsky JM, Snodgrass RG, Ono-Moore KD, Schneider DA, Newman JW, Adams SH, Hwang DH (2012). Saturated fatty acids activate TLR-mediated proinflammatory signaling pathways. J Lipid Res.

[67] Deczkowska A, Keren-Shaul H, Weiner A, Colonna M, Schwartz M, Amit I (2018). Disease-associated microglia: a universal immune sensor of neurodegeneration. Cell..

[68] Konishi H, Kiyama H (2018). Microglial TREM2/DAP12 signaling: a double-edged sword in neural diseases. Front Cell Neurosci.

