# Supplementary material for: GPR34 in spinal microglia exacerbates neuropathic pain in mice
Source: J Neuroinflammation. 2019 Apr 11;16:82. doi: 10.1186/s12974-019-1458-8 (PMC6458787; doi:10.1186/s12974-019-1458-8)
Supplement: Supplementary file 1 — Figure S2. Pain sensitivity is normal in control groups. PWT was measured in non-operated mice (Naive), sham-operated mice (Sham), and L4 nerve operated mice 0 (Pre), 3 and 7 days after injury (n = 4). ***p<0.001 (one-way RM ANOVA with Bonferroni adjustment or the nonparametric RM ANOVA on ranks with Dunnett’s adjustment). PWT of sham-operated mice and the contralateral paw of operated mice (Contra) was equivalent to that of naive mice. In contrast, PWT was significantly decreased in the ipsilateral side of operated mice (Ipsi). (DOCX 48 kb) [file 12974_2019_1458_MOESM1_ESM.docx]

**Additional File 1**

**

Figure S1**

**Pain sensitivity is normal in control groups.**

PWT was measured in non-operated mice (Naive), sham-operated mice (Sham), and L4 nerve operated mice 0 (Pre), 3 and 7 days after injury (*n* = 4). ***p＜0.001 (one-way RM ANOVA with Bonferroni adjustment or the nonparametric RM ANOVA on ranks with Dunnett's adjustment). PWT of sham-operated mice and the contralateral paw of operated mice (Contra) was equivalent to that of naive mice. In contrast, PWT was significantly decreased in the ipsilateral side of operated mice (Ipsi).
