# Supplementary material for: GPR34 in spinal microglia exacerbates neuropathic pain in mice
Source: J Neuroinflammation. 2019 Apr 11;16:82. doi: 10.1186/s12974-019-1458-8 (PMC6458787; doi:10.1186/s12974-019-1458-8)
Supplement: Supplementary file 2 — Figure S1. Microglial activation does not occur in control groups. Dorsal horn tissues at the L4 level were obtained 7 days after injury (n = 4), and mRNA expression of a microglial marker, Iba1 (a), and a prominently upregulated cytokine at day 7, IL-1β (b), was analyzed by qRT-PCR. Results are normalized to GAPDH and shown as fold change over naive sample. Expression of both molecules in sham-operated mice (Sham) and the contralateral side of operated mice (Contra) was equivalent to that in non-operated mice (Naive). In contrast, expression of both molecules was significantly increased in the ipsilateral side of operated mice (Ipsi). Values are mean ± SEM. **p < 0.01, ***p < 0.001 (one-way ANOVA with post hoc Turkey’s test). (DOCX 51 kb) [file 12974_2019_1458_MOESM2_ESM.docx]

**

Additional File 2**

**Figure S2**

**Microglial activation does not occur in control groups.**

Dorsal horn tissues at the L4 level were obtained 7 days after injury (*n* = 4), and mRNA expression of a microglial marker, Iba1 (a), and a prominently upregulated cytokine at day 7, IL-1β (b), was analyzed by qRT-PCR. Results are normalized to GAPDH and shown as fold change over naive sample. Expression of both molecules in sham-operated mice (Sham) and the contralateral side of operated mice (Contra) was equivalent to that in non-operated mice (Naive). In contrast, expression of both molecules was significantly increased in the ipsilateral side of operated mice (Ipsi). Values are mean ± SEM. ***p* < 0.01, ****p* < 0.001 (one-way ANOVA with *post hoc* Turkey’s test).
