# Supplementary material for: GPR34 in spinal microglia exacerbates neuropathic pain in mice
Source: J Neuroinflammation. 2019 Apr 11;16:82. doi: 10.1186/s12974-019-1458-8 (PMC6458787; doi:10.1186/s12974-019-1458-8)
Supplement: Supplementary file 3 — Figure S3. GPR34 mRNA is not detected in GPR34-deficient mice. Dorsal horn tissues at the L4 level were obtained from non-operated WT and GPR34-deficient mice (n = 4), and mRNA expression of GPR34 was analyzed by qRT-PCR. Results are normalized to GAPDH. Data are shown as fold change over WT sample. Values are mean ± SEM. ***p<0.001 (one-way ANOVA with post hoc Turkey’s test). (DOCX 40 kb) [file 12974_2019_1458_MOESM3_ESM.docx]

**Additional File 3**

**

Figure S3**

**GPR34 mRNA is not detected in GPR34-deficient mice.**

Dorsal horn tissues at the L4 level were obtained from non-operated WT and GPR34-deficient mice (*n* = 4), and mRNA expression of GPR34 was analyzed by qRT-PCR. Results are normalized to GAPDH. Data are shown as fold change over WT sample. Values are mean ± SEM. ***p＜0.001 (one-way ANOVA with *post hoc* Turkey’s test).
