# Supplementary material for: GPR34 in spinal microglia exacerbates neuropathic pain in mice
Source: J Neuroinflammation. 2019 Apr 11;16:82. doi: 10.1186/s12974-019-1458-8 (PMC6458787; doi:10.1186/s12974-019-1458-8)
Supplement: Supplementary file 4 — Figure S4. The dorsal horn contains LysoPS species. Quantification of representative LysoPS species (18:0-LysoPS, 18:1-LysoPS, 22:6-LysoPS) in the contralateral (Contra) and ipsilateral (Ipsi) dorsal horn 7 days after injury, using LC–MS/MS (n = 3). Contralateral side was used as a control. Amounts of 18:0-LysoPS and 18:1-LysoPS tended to increase in the injured dorsal horn, although the increases were not significant (one-way ANOVA with post hoc Tukey’s test). (DOCX 67 kb) [file 12974_2019_1458_MOESM4_ESM.docx]

**Additional file 4**

**

Figure S4**

**The dorsal horn contains LysoPS species.**

Quantification of representative LysoPS species (18:0-LysoPS, 18:1-LysoPS, 22:6-LysoPS) in the contralateral (Contra) and ipsilateral (Ipsi) dorsal horn 7 days after injury, using LC–MS/MS (*n* = 3). Contralateral side was used as a control. Amounts of 18:0-LysoPS and 18:1-LysoPS tended to increase in the injured dorsal horn, although the increases were not significant (one-way ANOVA with *post hoc* Tukey’s test).
